# A semantic similarity analysis of multiple English translations of *The Analects*: Based on a natural language processing algorithm

**DOI:** 10.3389/fpsyg.2022.992890

**Published:** 2022-11-10

**Authors:** Liwei Yang, Guijun Zhou

**Affiliations:** School of Foreign Languages, Northeast Normal University, Changchun, Jilin, China

**Keywords:** *The Analects*, translation, semantic similarity, readers, natural language processing

## Abstract

Working from the readers’ perspective, this study first investigates the online acceptance of the complete English translations of *The Analects* by investigating the number of online comments, downloads, academic citations, and other factors, and it ranks the different English versions according to how well they are received. The complete English translations of *The Analects* by D. C. Lau, James Legge, and 15 other translators are found to be well received by readers on mainstream online platforms. Then, based on five natural language processing (NLP) algorithms (TF-IDF, Word2Vec, GloVe, BERT, and SimHash), the 15 well-received English versions of *The Analects* are taken as samples to calculate semantic similarity. By comparing the semantic differences among the texts, this study analyzes the factors that affect the diversification of translated texts. (1) The influence of Chinese annotation on the translation semantics is great, even the greatest among many influential factors; and (2) different translators’ identities, the translation era, the translation purpose, and the translation background do not significantly affect the semantic influence of the translation. On the one hand, the readers can understand the differences between the different translations and choose an appropriate translation for their reading and learning more effectively. On the other hand, using the algorithms of NLP, we focus on the semantic similarity of different English translations of *The Analects* and analyze them to show the semantic differences quantitatively, which makes the comparison more intuitive and efficiently. Such a quantitative presentation of the results draws scholars’ attention to the differences in the translations.

## Introduction

*The Analects* is a collection of Confucius’ quotations compiled by his disciples and re-disciples during the Chinese dynasties of Spring and Autumn. It is a complete collection of the political ideas, ethical thoughts, moral concepts and educational principles of Confucius and the Confucian school. The American scholar Emerson said, “Confucius is the center of Chinese culture and education, philosophical Washington (Chi-Yun, 1944).” Voltaire described Confucius as “the wise man of the East (Voltaire, 1924).” Former President of the United States Ronald Reagan said that Confucius’ noble deeds and great ethical and moral thoughts have influenced not only his compatriots but also all humankind. As a masterpiece of Confucianism, *The Analects* has become not only a spiritual treasure of Chinese culture but also a treasure of world culture. How to effectively translate and disseminate it into a universal language so that the whole world can share the essence of Confucius’ thoughts has become the dream of sinologists and translators of all generations.

To achieve a better understanding of English translations of the Chinese classic *The Analects*, one must look at both the translated texts and their translators. The translation and dissemination of *The Analects* is a unified body composed of the translators, the process of translation, translation strategies, and the readers, as well as the interaction process among all the elements, as they play a vital role as a bridge to intercultural communication.

In the digital era, the variety of social platforms has diversified how information is disseminated. People’s reading has become increasingly fragmented and digitized, and readers’ motivations to read *The Analects* have become more diverse. Relying on a single translation tends to obscure its individual, historical, and textual complexity. Different translations also refer to different versions of the Chinese annotation, which may produce specific differences in the comprehension of *The Analects* readers.

For traditional Chinese literature or Chinese classic texts, comparative analysis is one of the main methods used to study translation styles (Xu, 2011; Wang, 2012). Some scholars also use textual comparison to assess translation quality (Han et al., 2019) by visually showing readers the similarities and differences between two or more translations. The application of computer-assisted qualitative data analysis is not new. The first successful computer-assisted analysis occurred in the 1960s when a mainframe computer was used to count words and phrases (Popping, 2000). Since then, computer-assisted qualitative data analysis processes have continued to develop, and the field has been marked by frequent innovation. Computer-based text analysis can be considered “more objective” compared to manual text analysis systems (Tian and Stewart, 2005). The translator is responsible for the original text as well as the reader. Translations of classic Chinese texts often bear the mark of their time. Different historical periods and life contexts have an important influence on the formation and dissemination of canonical translations. Baker defines “translator style” as a thumbprint expressed through various linguistic and non-linguistic features (Baker, 2000). For any given translation object, different translated texts have their own unique styles. However, the methods currently used most often to analyze differences in the characteristics of translated texts involve analyses of the word frequency, sentence length, the ratio of type & token, and the translated text for research (Haan and Esch, 2007; Li et al., 2011; Zhu and Ren, 2016) or study by analyzing its linguistic and rhetorical aspects (Qian and Kaufer, 2017; Helberg et al., 2018). Few texts have been analyzed in terms of textual semantic similarity, which is especially true of Chinese canonical texts. Text similarity measurements have been used in extractive summarization (Aliguliyev, 2009), automatic evaluation of machine translations (Denkowski and Lavie, 2011), and text coherence testing (Crossley and McNamara, 2011). It is possible to evaluate the translated texts and examine the translators’ translation characteristics by analyzing the texts’ semantic similarities.

In recent years, English translations of *The Analects* have been more widely disseminated in English-speaking countries. There seems to be a fashion among the public for saying, “The Master said” or “Confucius said.” For example, if we search for “the master said” or “Confucius said” on Twitter, Facebook, or Tik Tok, the results will include many quotations from *The Analects* commenting on or describing life and social phenomena. Following this, there will be many users liking, commenting, and retweeting. It is also interesting to note that the results will also include cases in which “the master said” or “Confucius said” was used as the beginning of a tweet or title but followed by a statement that is not from *The Analects*. It seems that adding “the master said” or “Confucius said” has become a way of emphasizing what internet users have to say, with a view to attract more attention to their statements.

*The Analects* is still very widely known and influential among general readers in English-speaking countries, which means English translations of *The Analects* are a valid object of study. By reviewing a large amount of information and browsing book websites or book sales platforms, one can see that there have been at least 110 different English translations of *The Analects* published from Randal Taylor publishing *The Morals of Confucius: A Chinese Philosopher* in 1691, to Australian scholar Victor Petersen publishing *Lunyu Analysis: The Analects or Sayings of Confucius* in 2021. The fact that many translators have been involved in translating *The Analects*, that means readers have more translations to choose from, but it also results in “pleasant perplexity” for general readers who do not know much about *The Analects* regarding which translation they should choose to read. Most readers are also unaware of the differences and connections between the many translations, let alone how to choose the right one to understand the entire text, a fragment, or even a classic quote from *The Analects*. Therefore, this study focuses on English translations of *The Analects* that are highly acceptable to readers and analyzes and summarizes the reasons for the differences between the different translations by comparing their semantic similarities.

Hans Robert Jauss and Wolfgang Ise founded the Reception Theory, also known as Reception Aesthetics. The theory emphasizes “acceptance” and considers the readers as the main role in the reading process. According to the theory, in the study, “acceptance” refers to the activity of both the ordinary and the professional readers in reading the texts, appreciating responses, extensive comments and depth interpretation, etc., in the spreading process of a book. This study uses Python web crawler technology to obtain the readers’ acceptability of each English translation of *The Analects* through mainstream websites based on the number of comments, downloads, literature citations, and the other factors we can obtain, and screen out 15 English translations of *The Analects* with high acceptability in mainstream websites as research samples, and then build a semantic similarity calculation framework based on classical semantic similarity algorithms: TF-IDF, Word2Vec, GloVe, BERT, and SimHash. We then use Python to establish the framework of text semantic similarity computation, and the computation logic of the five methods is similar: (1) text preprocessing; (2) import Python modules (such as pandas, NumPy, gensim); (3) import preprocessed texts 15 English translations of *The Analects*; (4) import the word vector models corresponding to each algorithm; (5) similarity calculation; (6) export results to excel files and close the program. With the computed data derived from the above logic, the differences existing in each translation are presented in the data along with the differences in each text, and the reasons for the differences in each translation are analyzed from a macro perspective. The results help readers understand the differences between the different translations more easily and choose a suitable translated text for their reading and learning. This study also provides some suggestions for translators who will work on *The Analects*, drawing on previous translations of the classics.

## Materials and methods

### Study sample selection

With the development of computer-aided technologies, more readers read with the help of electronic products (e.g., mobile phones and Kindles). Readers’ reading habits have shifted from paper books to a combination of e-books and paper books. Although online data collection cannot fully reflect the acceptance of translations, there is no doubt that data on the reading of e-books and some printed books can reflect readers’ reading preferences and attitudes toward the book quality. The author obtained around 110 English translations of *The Analects* through Internet research, including the platforms used for this research (listed below), other Internet platforms, and libraries. Most of these translations can be read or obtained free of charge on Amazon, Archive.com, PDF Drive, and other websites or libraries. Although there is a fee to access some translations to cover copyright and platform operation costs, they are not as expensive as most commercial books, costing about the same as a McDonald’s hamburger. Therefore, for readers, cost has little influence on their choice of translation. In terms of how to measure popularity with data, the number of comments is an important indicator of reader acceptance of a translation. In the comments, positive evaluations tend to be far more numerous than negative ones, even considering negative comments about issues external to the translation, such as printing quality or the delivery of non-textual elements. Therefore, it is feasible to take the number of comments as an important indicator of attention. The texts in this study are only used to obtain a sample to measure the semantic similarity between the various English versions of *The Analects*.

Based on Amazon (reviews), Goodreads (ratings + reviews), Archive.com (views), Google Scholar (times cited), and PDF Drive (times download), “Confucius” and “Analects” were used as search keywords, and a Python (Python3.9) program was used to crawl each website. The number of comments, downloads, times read, and other relevant data on the different English versions of *The Analects* are given in Table 1.

**Table 1:** Number of hits from the original data and the valid data.

Search keyword	Amazon	PDF drive	Good reads	Google scholar	Archive
**Original data**					
Confucius	990	1,140	1,284	1,786	328
Analects	275	443	198	654	97
**Valid data**					
Confucius/analects	109	161	272	52	124

Original data contains all the results of the search keywords, including the translations of *The Analects* by each translator, but also many irrelevant results that need to be filtered.

The filtering criteria are as follow:

1. The retained data contain a translation with a clear translator’s name and are sorted based on the year of publication
2. If the translator’s name is unclear, the authors used the fetching book links to find the e-book, checked the information on the cover, and preview the content available for downloading. If there is not enough information, the authors pay to obtain a version with the translator information, adding it to the list and sorting it by year
3. Books not related to the English translation of *The Analects* (generated by a fuzzy search on the website) are directly deleted and excluded from the research field.

After further processing of the valid data above and grouping together versions by the same translator that appears among the five websites above, there are 66 apparent English translations of *The Analects*. The following data do not indicate that a particular translation appears only once on a website. For example, James Legge’s translation has 25 different reprints and 25 purchase links on Amazon. We list the number of comments on these 25 versions after combining the number of comments, and this represents Legge’s data; the same applies to other translators and websites.

Based on the volume and the frequency of data across the five websites here, we define *i* as the frequency of the translation appearing on the websites of the five platforms and take the data when *i* ≥ 3. This takes into account the equilibrium of the data. In other words, the versions appear more than three times on the five websites for 25 translations are given in Table 2.

**Table 2:** Data for English translations of *The Analects* appearing in three or more of the five investigated websites.

SN.	Translator	Pub year	Amazon	Goodreads	Archive	Google	PDF drive
	*i* = 5						
1	J. Legge	1861	1,028	973	35,176	43	11,399
2	W. Jennings	1895	910	3	8,958	238	1,918
3	D. C. Lau	1979	923	20,889	390	768	1,545
4	E. Pound	1951	34	524	715	9	8
	*i* = 4						
5	R. Ames	1998	138	0	69	1,221	621
6	A. Waley	1938	74	67	7,513	17	0
7	L. Lyall	1909	40	121	7,292	0	61
8	Lin Yutang	1938	20	163	6,854	7	0
9	Annping Chin	2014	14	31	505	0	791
10	B. Watson	2007	16	0	40	34	2,995
11	Bruce Brooks	2004	9	0	7	482	1,128
12	David Hinton	1998	4	203	131	10	0
13	David L. Hall	1999	2	25	83	1,309	0
14	Van Norden	2003	1	12	0	156	39
15	E. Slingerland	2003	0	52	29	504	1,316
	*i* = 3						
16	Lionel Giles	1907	31	93	7,609	0	0
17	T. Cleary	1992	20	253	0	0	1
18	Peimin Ni	2017	12	20	0	140	0
19	R. Dawson	1993	6	17	167	0	0
20	J. R. Ware	1959	4	47	187	0	0
21	Adam Sia	1997	2	1	177	0	0
22	Ku Hung-Ming	1898	0	11	3,437	10	0
23	H. Rosemont	2013	0	9	0	37	158
24	R. Eno	2005	0	0	3,680	5	276
25	J. Marshman	1809	1	0	427	0	59

We first calculate the proportion of attention of each translator’s translation on the corresponding network platform and can see that the calculated percentage reflects the attention it attracts to the platform to a certain extent. Then, we calculate each translator’s average proportion of attention on the five network platforms to make a comprehensive ranking. This study takes the complete translation of *The Analects* as the research object, so we ruled out the abridged translations of Roger T. Ames, David L. Hall, E. Bruce Brooks, Lionel Giles, Lin Yutang, Van Norden, Thomas Cleary, Henry Rosemont, Marshman, and Adam Sia. The ranking of the 15 complete translations in terms of the attention they attract are given in Table 3.

**Table 3:** Comprehensive acceptability ranking of English versions of *The Analects.*

SN.	Translator	Amazon	Goodreads	Archive	Google scholar	PDF drive	AVG
% Percentage							
1	D. C. Lau	26.25	85.05	0.32	14.96	6.86	26.69
2	James Legge	29.24	3.96	28.99	0.84	50.64	22.73
3	W. Jennings	26.82	0.01	7.38	4.64	8.52	9.52
4	E. Slingerland	0.00	0.21	0.02	9.82	5.85	3.18
5	B. Watson	0.46	0.00	0.03	0.66	13.31	2.89
6	A. Waley	2.10	0.27	6.19	0.33	0.00	1.78
7	L. A. Lyall	1.14	0.49	6.01	0.00	0.27	1.58
8	Annping Chin	0.40	0.13	0.42	0.00	3.51	0.89
9	R. Eno	0.00	0.00	3.03	0.10	1.23	0.87
10	E. Pound	0.97	2.13	0.59	0.18	0.04	0.78
11	Peimin Ni	0.34	0.08	0.00	2.73	0.00	0.63
12	Ku H. M.	0.00	0.04	2.83	0.19	0.00	0.61
13	D. Hinton	0.11	0.83	0.11	0.19	0.00	0.25
14	J. R. Ware	0.11	0.19	0.15	0.00	0.00	0.09
15	R. Dawson	0.17	0.07	0.14	0.00	0.00	0.08

For example, the number of reviews for D. C. Lau’s translation on Amazon is 923, whereas the total number of reviews for *The Analects* on Amazon is 3,516, which gives 923/3516 (equal to 26.25%). The same is calculated for other translations and websites; the last column is the average percentage among the five websites as the basis for ranking.

The translators are divided into three major categories according to their nationalities and educational backgrounds: (1) translators whose native language is English, referred to as Western translators; (2) translators who are Chinese but whose education is mainly Western, referred to as foreign-Chinese translators; and (3) translators who have received a traditional Chinese education, referred to as Chinese translators. The above table shows that English translations of *The Analects* by Western translators are generally well received on online platforms. D.C. Lau was born in Hong Kong, China, and studied and taught in England for a long time. Peimin Ni received his traditional Chinese education until he graduated from Fudan University. He went to the University of Connecticut in the United States for further study and worked in American universities ever since. Annping Chin moved to the United States at the age of 12 and received an American education. Ku Hung-Ming received a Western-style education until he moved to China at the age of 23 and only studied Chinese culture extensively after he returned to China. The above four translators can be described as foreign-Chinese translators. Other examples of this include Tehyi Hsieh, Wing-tsit Chan, Chichung Huang, and Tom Te-wu Ma. When these translators translated *The Analects*, they did so in the context of a more Western education. As for Chinese translators in the traditional sense, about 50 translators have translated *The Analects* since Lin Yutang, but few editions have been sold on Western online platforms or have only appeared occasionally, and their reception figures are not high. Yu Dan’s text attracted 67 reviews on Amazon and 640 comments and reviews on Goodreads, which is quite a lot of data, but it returns no results on “Internet Archive Views,” “Google Scholar Cited,” or “PDF Drive.” Fu Yuhua’s text was only viewed 541 times according to “Internet Archive Views,” and no data about it were available from the other Internet platforms.

### Text preprocessing

**Figure 1:**
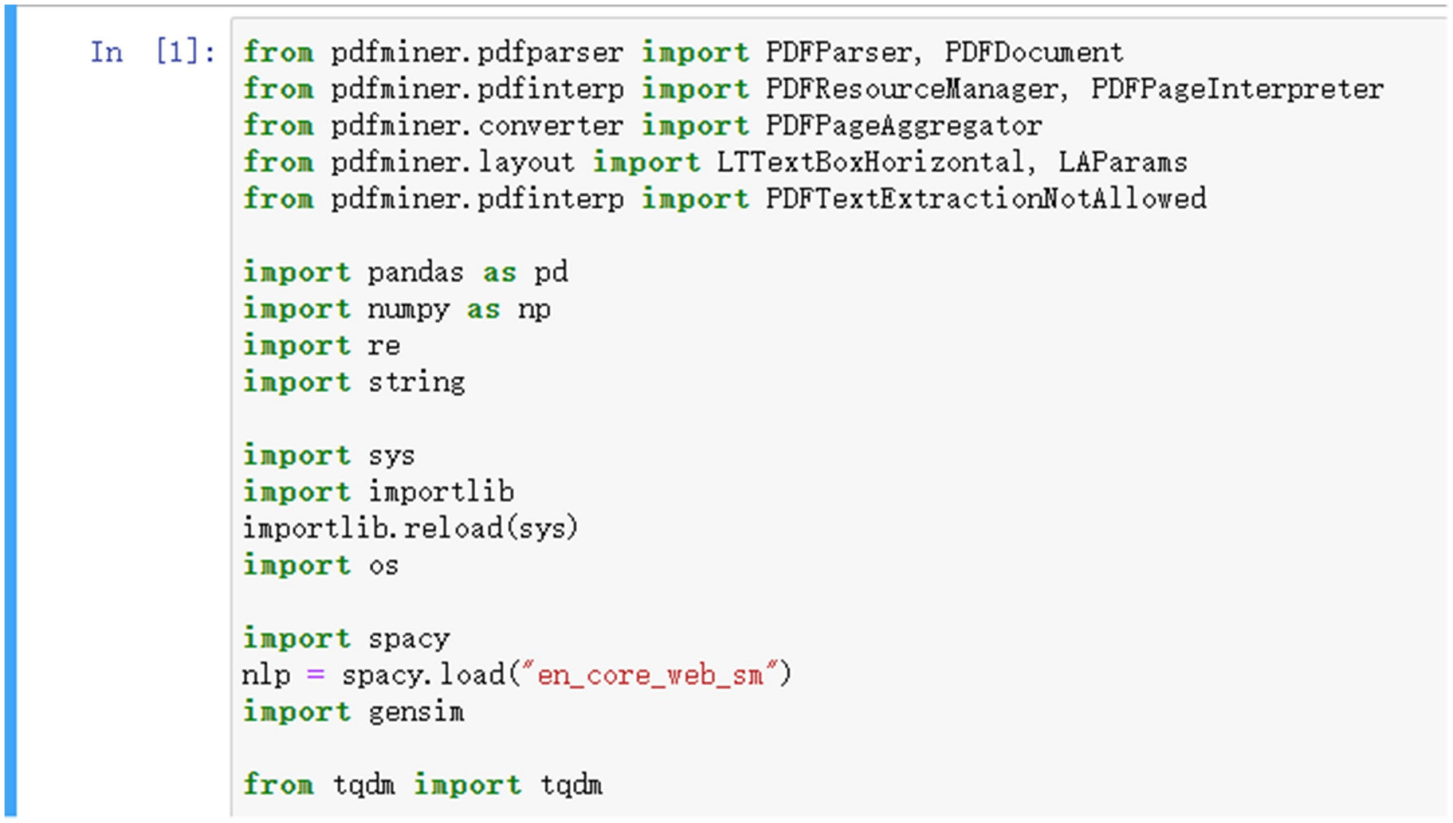
Importing the Python modules required for text preprocessing.

Statistical machine learning approaches are currently the mainstream of research in the field of natural language processing (NLP). These approaches usually automatically or semi-automatically acquire statistical knowledge of language from training data and can effectively build a representation model of language. However, statistical machine-learning-based methods rely heavily on the training data size, representativeness, correctness, and processing depth, and the more linguistic data and the stronger the domain of the training data, the better the fit of the language model. Based on the analysis in the previous section, we first selected the top 15 English translations of *The Analects* that had achieved high acceptability on the online platforms and used the “Jupyter Notebook (6.4.12)” development tool in Python to complete the training of word vectors in the corpus as a model for subsequent analysis. The Python modules are as follows (the detailed program has been uploaded to figshare; Figure 1).

**Figure 2:**
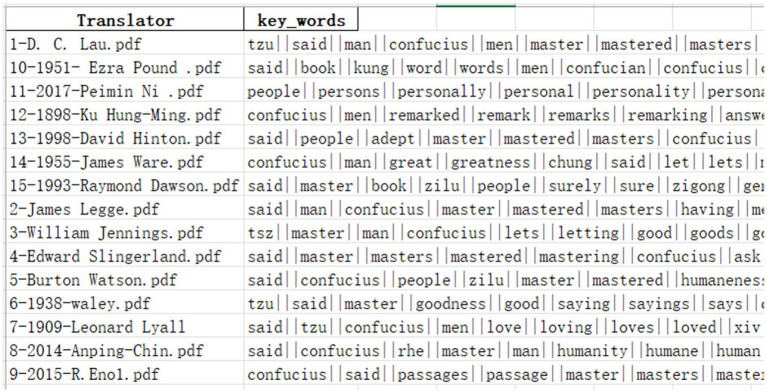
Examples of keyword features in different translations.

The text needed to undergo a series of text preprocessing steps, including spell checking, tokenization, and normalization. Not all preprocessing steps need to be checked and fulfilled, as this depends on the specific corpus form (Han et al., 2021). As we obtained the English translations of *The Analects* in high-resolution PDF versions, they could be recognized directly in Python without secondary conversion. Then, we set the preprocessing of data, such as the deletion of special symbols, directly reading the PDF file in a loop, retaining the English text, converting capitalized words to lowercase, removing special characters, stop-words, and other operations. We identified the words in the PDF text and conducted further noise reduction and filtering operations. We then used the Python classic module gensim algorithm to extract keywords from the object text. In a gensim keyword extraction, typically 100 to 500 words are used to identify the significant features of the article. Here, for more accurate analysis, by the keyword weights calculated by gensim, we stored the first 1,000 keywords for each translation in Excel as training data to facilitate the subsequent use of each algorithm for comparative text analysis. An example of the trained lexicon model is as follows (Figure 2).

### Introduction of the NLP algorithm

Text mining has been applied to a variety of research areas. These include text classification, clustering, opinion mining, and information extraction and retrieval. In all these areas, measuring the degree of textual similarity is essential for identifying semantic relationships among texts (Cho and Kim, 2017). Here, we chose five algorithms, TF-IDF, Word2Vec (Mikolov et al., 2013), GloVe (Pennington et al., 2014), BERT (Devlin et al., 2019; Alammar, 2020), and SimHash (Manku et al., 2007), for NLP to conduct a similarity study of English translations of *The Analects*.

TF-IDF (Term Frequency-Inverse Document Frequency), also known as word frequency-inverse document frequency, is a statistical method used to evaluate the importance of words for a document set or one of the documents in a corpus:

$TF_{i,j}=\frac{n_{i,j}}{\sum_{k}n_{k,j}}$

$IDF_{i}=\log\frac{\left|D\right|}{1+\left|j:t_{i}\in d_{j}\right|}$ where *n_i,j_* indicates a term *t_i_* number of occurrences in document 𝑑_𝑗_, and *TF_i,j_* indicates the frequency of word *t_i_* in document *d_j_*. |D| indicates the number of all documents, and |*j: t_i_* ϵ *d_j_*| indicates the number of documents containing the word *t_j_*, we added 1 to this value the number of entries containing *t_i_* from being 0, which could cause an error. TF-IDF is expressed as:

TF−IDF=TF∗IDF .

To summarize, the more times a word appears in an article, the more representative it is of that article.

The Word2Vec algorithm was proposed by Google engineers Tomas Mikolov et al. in 2013 (Mikolov et al., 2013) and is a model to learn semantic knowledge from a large corpus of text in an unsupervised way, which is widely used in NLP. Word2Vec is a lightweight neural network whose model only includes an input layer, a hidden layer, and an output layer. The model framework mainly includes the CBOW and Skip-gram models, according to the input and output. The word vectors learned by Word2Vec represent the semantics of words and can be used for classification, clustering, and word similarity calculation. The vectors generated by Word2Vec are directly used as the input of the deep neural network, which can be used for work such as sentiment analysis.

After the introduction of Word2Vec, the GloVe (Global Vector) algorithm was proposed (Pennington et al., 2014). Traditionally, there are two main approaches to implementing word embedding, Matrix Factorization Methods and Shallow Window-Based Methods, and both have their respective advantages and disadvantages. At the same time GloVe combines the advantages of both. The experiments in the paper by Pennington and colleagues show that GloVe methods are superior to methods such as Word2Vec.

BERT (Bidirectional Encoder Representation from Transformers) is a pre-trained language representation model. The structure of earlier pre-trained models was limited by the unidirectional language model (left-to-right or right-to-left), thus limiting the model’s power to represent unidirectional contextual information. BERT uses masked language model (MLM) pre-training and a deep bidirectional transformer component to build the entire model, thus generating a deep bidirectional language representation that incorporates left and right contextual information. It emphasizes the use of the new MLM to generate deep bidirectional language representations instead of a traditional unidirectional language model or the shallow splicing of two unidirectional language models for pre-training, as in the past. The BERT paper mentions that new state-of-the-art results were obtained in 11 NLP tasks. Thus, the BERT algorithm is one of the most comprehensive algorithms available.

In tasks such as text classification, clustering, similarity calculation, we want to represent the text with a fixed-length numerical vector, as this allows us to calculate text similarity using methods such as Euclidean distance. Common textual representation models include TF (Term Frequency), TF-IDF (Term Frequency-Inverse Document Frequency), and sentence vectors. In a massive text de-duplication scenario, the above models have some limitations: (1) The TF accuracy is low, namely, it is not very sensitive to the assessment of the similarity range from “very similar” to “identical”; it is influenced by the length of the text and other factors, and the similarity threshold is difficult to set. (2) TF-IDF requires first traversing all the texts to get the IDF, which is more computational; (3) Sentence vector is mainly concerned with semantics and is not good at identifying literal similarity; it is more computational. SimHash overcomes the shortcomings of the above algorithms and can also be seen as a distributed representation of the text. Of course, the ability of SimHash to mine text information is not as strong as that of a model such as Word2Vec. SimHash encoding will set an upper limit on the effect of classification and clustering models. In general, in a low-precision, high-speed scenario, SimHash is worth trying. Hamming Distance is the classic partner of SimHash and is used to measure the distance or similarity between texts based on SimHash code. Suppose there are two data strings of equal length (length = *k*):

$A=\left(a_{1},a_{2},\ldots \ldots ,a_{k},\ldots \ldots ,a_{k}\right)$

$B=\left(b_{1},b_{2},\ldots \ldots ,b_{k},\ldots \ldots ,b_{k}\right)$

$Hamming\;Distance=\overset{k}{\underset{k=1}{\sum }}s_{k},$

$s_{k}=\{\begin{array}{l} 0,if:a_{k}=b_{k}\\ 1,if:a_{k}\neq b_{k} \end{array}$

NLP-related tasks in which natural language is to be given to algorithms in machine learning usually require the mathematization of the language because machines are not humans and only recognize mathematical symbols. A word vector is a way of mathematicising words in a language, and, as the name implies, a word vector is a representation of a word as a vector. As is well known, words need to be encoded into numerical variables before they are sent to neural networks for training. Four algorithms – TF-IDF, Word2Vec, BERT, and GloVe – are used for these keywords, which convert them into word vectors that aggregate into sentence vectors (high-dimensional word vectors containing semantic information) that represent the topic of the article. With these document feature vectors, the similarity of the articles is obtained by calculating the cosine similarity two by two. Regarding the choice of vectors, Word2Vec uses Google News, a 300-dimensional model open-sourced by Google. GloVe and BERT use Von Platen’s open-source pre-training model. The SimHash algorithm is different from the above four algorithms in that it calculates the similarity between different English versions by hamming distance.

### Similarity calculation

**Figure 3:**
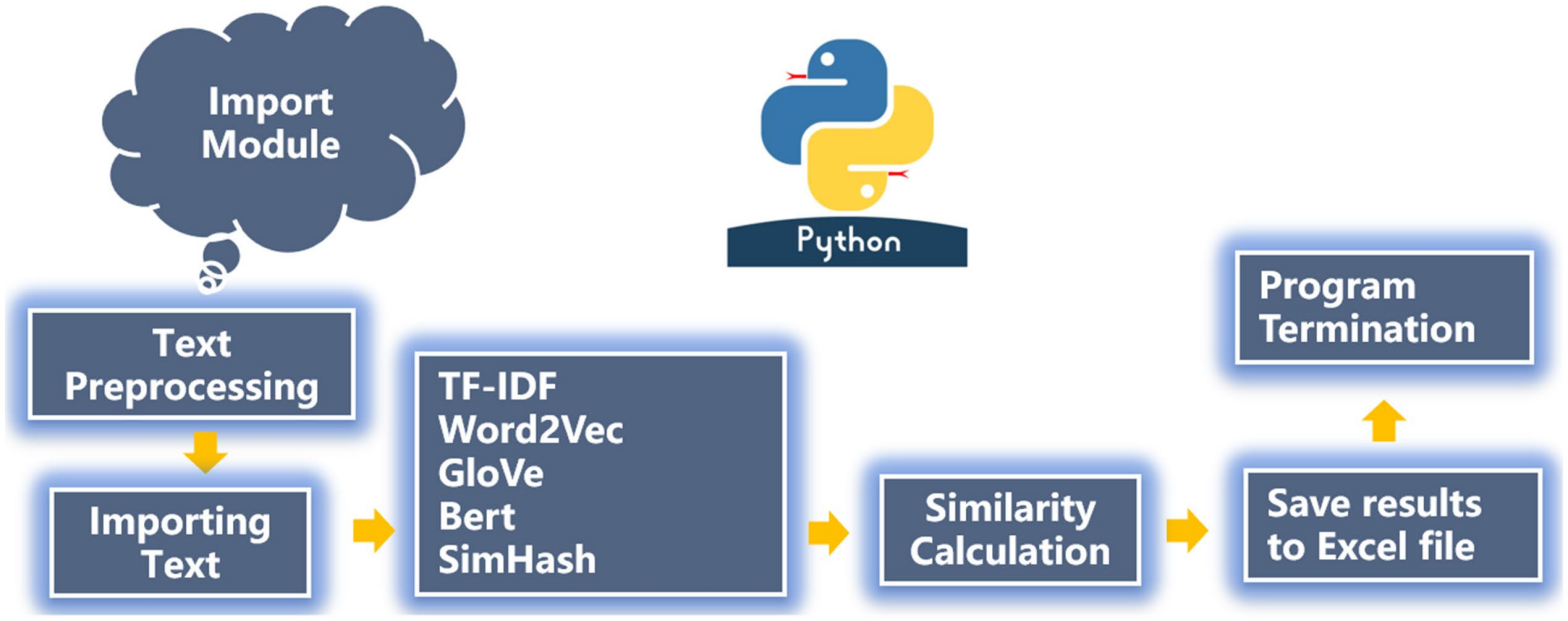
Logic diagram of text similarity calculation by Python.

For TF-IDF, we can directly use the logic below to calculate the text similarity, whereas for the Word2Vec algorithm, we need to import the GoogleNews-vectors-negative300.bin word vector model before calculating the similarity. For the GloVe and BERT algorithms, we choose the bert-large-uncased open-source pre-trained word vector models “glove.6B.300d” and “bert-large-uncased” (Von Platen, 2019). The SimHash algorithm is different from Word2Vec, GloVe and BERT, because it can directly drag in the data model and also calculate text similarity through its logic of data preprocessing, reading files, text filtering, calculating similarity, and results-saving (Figure 3).

## Results

**Figure 4:**
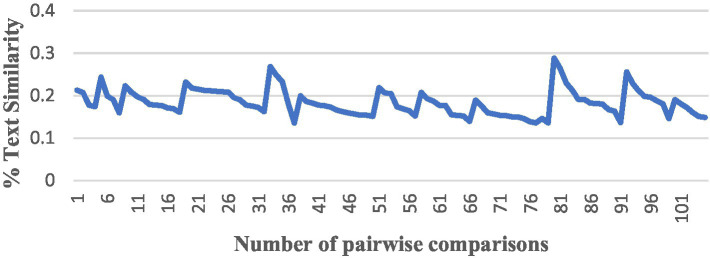
A comparison by TF-IDF.

**Figure 5:**
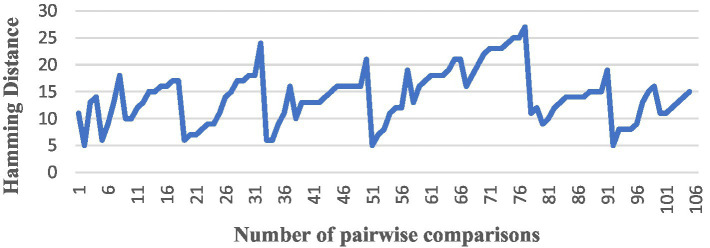
A comparison by SimHash.

**Figure 6:**
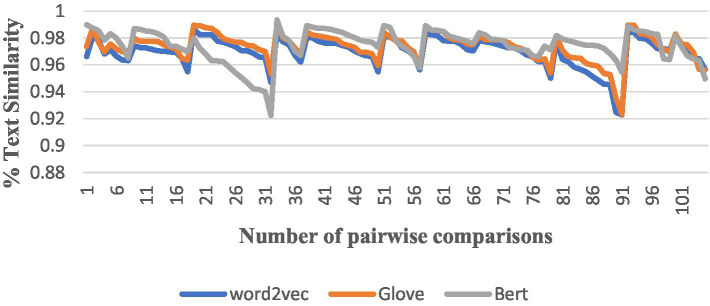
Comparisons by Word2Vec, GloVe, and BERT.

After completing the above preparation, we started to conduct a comparison of the similarity between pairs among the 15 English translations of *The Analects*, and we used all the algorithms to perform the pairwise comparison, producing a total of 105 results, and numbered the results of each version pair comparison from the NO. 1 to the NO. 105. The trend in the similarity results for each algorithm was as follows (Figures 4–6).

The vertical axis is the interval range of text similarity, and the horizontal axis is the data location numbers of the 15 translations compared in pairs, from 1st to 105th.

### Analysis of calculation

We can see that the results of comparing the semantic similarity of different English texts of *The Analects* using the TF-IDF algorithm are between 13 and 29%, and the Hamming distance of the SimHash algorithm is between 5 and 27. The three algorithms for text semantic similarity comparison (Word2Vec, GloVe, and BERT) returned results above 92%, and the curve trends of the corresponding positions were the same, which proves that the semantic calculation accuracy of these three currently used algorithms is also high.

The meaning expressed in the original Chinese text of *The Analects* should result in a high level of similarity between the semantics of the different English translations. However, there are large deviations between the above TF-IDF and SimHash algorithms. In a comprehensive analysis, the author believes that such deviations are mainly due to: (1) inconsistencies in the versions of the annotations used by each translator, as well as the translation strategies of each translator, translation level, and identity, and (2) the algorithm itself. The traditional TF-IDF algorithm only measures the importance of words in terms of “word frequency” and the subsequent sequence of feature values of documents, which are independent and cannot reflect the sequence information. The algorithm is easily affected by the skew of the data set, such as a large number of documents in a certain category, which leads to the underestimation of IDF. IDF improvement algorithms such as TFIDF-FL (Zhang et al., 2019) have been proposed, and some scholars have also suggested combining TF-IDF with Word2Vec to solve the shortcomings of TF-IDF (Naeem et al., 2022); in short, simply using the TF-IDF algorithm to calculate semantic similarity leads to the problem of low accuracy.

While the features of the SimHash algorithm are as mentioned above, its text similarity calculation is suitable for low-precision and high-speed scenarios. This calculation has lower requirements for speed but higher requirements for accuracy, which proves that SimHash is unsuitable for studying long texts or for high-precision similarity calculations.

Meanwhile, although the numerical results of the two algorithms TF-IDF and SimHash deviate slightly from those of algorithms and objective reality, there is consistency in the overall curve direction when comparing their result trend graphs, as well as when comparing the result trend with the three algorithms Word2Vec, GloVe, and BERT, which objectively proves that the different translators’ versions of *The Analects* still somewhat differ in semantics.

### Analysis of high-similarity versions

We selected several versions that were shown to be closer in similarity through the Word2Vec, GloVe, and BERT algorithms for analysis.

For the versions of Ezra Pound (Confucius, 1969) and James Legge (Confucius, 1861), the calculated similarities are 97.618, 97.972, and 98.647%, which are all at a high level. Reading Ezra Pound’s translation, we find that James Legge is mentioned five times in the translation notes, with the intention to explain the plasticity of Legge’s translation at some point. However, it can still be seen that Pound’s translation is still deeply influenced by Legge’s translation, although nearly a century had passed.

Ezra Pound lived primarily in the first half of the 20^th^ century, which experienced two world wars and the post-war recovery process. Ezra Pound argued that the existing translations of *The Analects*, while providing a complete translation of the source text, have not been thought about and presented by the translator from the original author’s perspective (Confucius, 1969). Faced with this historical context and situation, he translated *The Analects*, and his translation was not well received by the translation community from the perspective of traditional translation theories (Zikpi, 2018). However, an analysis of the translation’s attention data performance showed that it was able to attract readers’ attention successfully. Amazon, Goodreads, Internet Archive, Google Scholar, and PDF Drive are the five sites where Pound’s translation can be found, with 715 views on Internet Archive and 524 readers in Goodreads participating in the rating and evaluation of his translation. The original text of *The Analects* is concise and easy to read and remember. Pound’s translation retains the style of the original text, making use of simple sentences and pursuing structural similarity with the original text in the form of couplets and prose. This reflects his translation’s characteristics of simplicity and economy, letting the characters speak with vigor, which greatly reduces the difficulty of reading. These factors may be why Pound’s translation has reached a certain level of acceptance among readers.

The calculated similarities between William Jennings (Confucius, 2010) and James Legge (Confucius, 1861) are 97.276, 97.788, and 97.368%, respectively, which are all at a high level. By reading William Jennings’ preface, we can find that his translation refers to James Legge’s lexical index. However, he also emphasizes that to be different from James Legge’s translation, on the basis of that, he gave his own translation and understanding. However, Jennings was still heavily influenced by Legge’s translation. In addition, like James Legge, William Jennings revered the importance of Confucianism in serving his missionary work better. However, his translation is not as obviously Christian as that of James Legge.

The English translation of William Jennings is not very well known in the field of *The Analects*, and few researchers have conducted scholarly studies based on his text. However, it still receives great attention among the five websites above, second only to the translations by D. C. Lau and James Legge. Through an analysis of his text, we found that William Jennings’ version has its unique features, especially in creative reorganization of content and structure, objective and detailed annotations, artistic rendering of culture-specific expressions and verses, as well as an individualized way of interpreting core concepts, which are all the results of the translator’s exercise of creativity and subjectivity. Jennings reached a balance between “loyalty” and “treason,” and he attached great importance to the exposition of the artistic qualities of the text and showed appreciation for the readers.

The calculated similarities between Leonard Lyall’s (Confucius, 2007) and Ku Hung-Ming’s (Confucius, 2011) versions are 97.262, 97.614, and 97.294%, whereas those between Leonard Lyall’s and James Legge’s versions are 95.638, 95.797, and 95.778%. In the preface to Lyall’s translation, it is stated that the notes and introductory sections of the translation are from James Legge, and the Chinese terms are based on Ku Hung-Ming’s English translation.

The English translation of *The Analects* by Leonard Lyall is positioned seventh in terms of acceptability and has appeared on four of the five aforementioned websites, particularly the Internet Archive, where it has been watched 7,513 times and has performed relatively well in terms of acceptance data. Leonard Lyall joined the Chinese customs service in 1886 as a “customs officer” and had lived and worked in China for more than 40 years. Leonard Lyall and Ku Hung-Ming had a close personal relationship, and they had a profound exchange and conversation about the English translation of *The Analects*. Leonard Lyall believed that James Legge’s translation had certain defects, so while referring to the James Legge’s translation, he improved the parts he considered to have translation defects. Leonard Lyall’s translation strategy tends toward a literal translation. It also follows the original text closer and effectively illustrates the essence of “authentic translation.”

### Analysis of low-similarity versions

The conclusions drawn by the three algorithms Word2Vec, GloVe, and BERT show that translations of *The Analects* by each translator show a semantic similarity in high-level segments. However, there is indeed still some semantic deviation.

The calculated similarities between the translations of Annping Chin (Confucius, 2014) and D. C. Lau (Confucius, 1979) are 94.679, 95.3, and 92.237%, which are relatively low. D. C. Lau found that the translations of some Chinese classics were not loyal enough, so he translated *The Analects* and other classics himself. The version by D. C. Lau refers to the Chinese annotations of Zhu Xi, He Yan, and Xing Bing, with some of his own opinions on *The Analects*, and simplifies some phrases. He used precise language to express clear concepts, and his translations were widely received and had the unique characteristics of his own. He used a literal translation, translating sentence by sentence and adjusting the word order. His translation strategies are flexible, and his language is highly acceptable to readers.

Ms. Annping Chin had an excellent background in sinology: her grandfather was famous historian Jin Yufu, her supervisor during her doctoral studies was well-known Ming and Qing historian Fang Zhaoying, and her husband was the famous contemporary American sinologist, Jonathan Spence. Her purpose in translating *The Analects* was that Western readers of the time considered the book as “indefinable in its meaning as Montaigne’s prose.” She believed that Western translations of *The Analects* at the time did not reflect its rich interpretive history, with one of the reasons being that most translators relied only on Zhu Xi’s Chinese annotation. She recognized that “the scholars of the last three centuries in China (especially Qing Confucianism) had effectively sought the truth, exhibiting multiplicity and diversity in their treatment of words, sentences, and passages, and that their translations aim to be clear and concise while at the same time scholarly and deep, hoping to engage readers through the chapters without losing them in the middle,” thus “making it possible for readers to engage in Confucius’ dialogs and to learn from them what they can use in their own lives” (Confucius, 2014). Therefore, she selected Cheng Shude’s Chinese annotation from the Qing Dynasty, showing great concern for exegesis and koan, focused on the translation of existing koan results, and tried to restore the historical and cultural context of the period when the text of *The Analects* was born in an interlingual manner. The translation shows the general characteristics of simplicity and fluency, and rich and careful annotation—increasing cultural communication and improving the acceptance of Western readers. Therefore, we can speculate that the obvious differences in the semantic similarity between the translations of Annping Chin and D. C. Lau could be mainly caused by their different choices of Chinese annotation.

The calculated similarities between the translations of Peimin Ni (Confucius, 2017) and Leonard Lyall (Confucius, 2007) are 92.274, 92.3, and 95.456%, which are relatively less similar. The purpose of Peimin Ni’s translation is to address how *The Analects* are not easy to understand and even more difficult to translate, and the current English translations are deficient. The text is often so vague for modern readers that it has little meaning or permits multiple and sometimes conflicting interpretations. Thus, it was decided to retranslate to reproduce the original text to be more faithful to the original work (Confucius, 2017). Peimin Ni’s translation is not based on single Chinese annotation. He says, “I mainly referred to the significant Chinese annotation by Zhu Xi, Huang Kan, and He Yan. Other enlightening Chinese annotations were also adopted, such as the interpretations of Kang Youwei and Li Zehou, which often reflect their responses to the historical periods in which they lived. I also referred to the Chinese annotation of some Japanese and Korean scholars and the results of contemporary Chinese and foreign academic research” (Ni and Tao, 2021). Creating a translation that is close to the original text is the main strategy of Peimin Ni’s English translation of *The Analects*, which reproduces the original rhetorical effects and stylistic features. As for Leonard Lyall’s translation, although the Chinese annotation is not mentioned among the notes of the translation, by analyzing the features of his translation, some scholars have proved that the Chinese annotation is Zhu Xi’s annotation and that he drew on the translations of both James Legge and Ku Hung-Ming. Leonard Lyall acknowledged the extensive notes of his predecessors for completing his translation and expressed his deep appreciation for James Legge’s translation. Although acknowledging that many of the notes and much of the introduction refer to James Legge’s views; at the same time, he notes that although the James Legge translation is valuable, it is flawed (Confucius, 2007). Leonard Lyall’s translation highlights the literal rendering and is closer to the original. In summary, we can see that the Chinese annotations chosen by Peimin Ni and Leonard Lyall are different.

(The detailed publication information of the English translations of *The Analects*, which we discussed above, is listed in the form entitled “Publication information of the English translations of *The Analects*” in figshare.)

## Discussion

*The Analects* has been translated into English for hundreds of years, with more than 100 translators involved. *The Analects* has become increasingly popular in English-speaking nations. In this study, Python crawler technology is used to demonstrate the reader acceptance of *The Analects*, and the efficiency and precision of data collection are significantly improved. Based on the data, we performed a macro-level analysis of the distribution of reader acceptance and selected 15 complete English translations of *The Analects* with high acceptability. The analysis indicates that Western translators and translations by Chinese translators with Western education receive more attention on the five websites listed above.

In the past, translation research in digital humanities has generally focused on statistics, word frequency, sentence length, and the ratio of type & token in the discourse, whereas very few have examined and evaluated translation outcomes at the semantic level. Contrarily, examining other textual elements does not immediately assist readers or scholars in comprehending the translation because the semantic level is significantly more important for understanding reading content than other textual features. Additionally, due to the differences between translations, readers still get confused about the whole or some phrases of *The Analects*, mainly in understanding at the semantic level. There is no scientific method to quantify these differences in translation research and visualize them.

Translations made at different times tend to be made under different conditions and turn out differently, not because they are good or bad but because they must be produced to satisfy different demands (Bassnett and Lefevere, 1990). This study uses the algorithm of NLP similarity calculation to extract critical information, summarizes the text semantic features through a complex semantic analysis, and quantifies the degree of semantic differences among translations for more intuitive comparisons. On the one hand, this method can assist researchers in discovering some unique features of specific translations, which greatly highlights the differences in translations and is conducive to further exploring the deep reasons for the semantic differences. On the other hand, the 15 versions chosen for this study have similarities ranging from 92 to 99%. We can speculate that the translations with higher acceptability all more effectively convey the semantics of the original text and enable readers to better understand the connotation of the ideas the author intended to convey. By comparing such differences between the English versions of *The Analects*, readers can select the appropriate translation for reading and learning. The author even recommends that, to prevent or minimize misunderstanding, native English speakers read *The Analects* based on at least two or more of the top 15 books in this study, as they will further benefit the readers through the reflection of the text on the philosophies of Confucius on being and doing oneself. At the same time, the use of several English versions strengthens the diffusion of this classic work from the readers’ point of view and improves its communication effect.

This study attempts to determine the reasons for the differences in English translations of *The Analects* from four aspects: the translator’s identity, the translation era, the Chinese annotation, and the purpose and characteristics of the translation. According to the final results of the analysis’ there are few significant and evident distinctions between translation eras and translators’ identities. These two points are chosen as factors for the study because some scholars believe that the differences in the translation era or the translator’s identity lead to different translation purposes, which affect the semantic representation of the translated texts. The author considers this opinion controversial in terms of semantic similarity. For example, Ezra Pound was an American poet and literary critic, and his translation was published in 1951. James Legge’s central identity is an English missionary and sinologist, and his translation was published in 1861. The two translations differ significantly in terms of the publication date and the translator’s identity, but their similarity is still high (97.618, 97.972, and 98.647%). We can also see that Annping Chin and Peimin Ni both received a traditional Chinese education as teenagers and later western-style education. However, when comparing the two English versions, the semantic similarity is 92.467, 93.498, and 96.357%, which is not exceptionally high. The semantic similarity between the versions of D. C. Lau and Annping Chin is also not exceptionally high at 94.679, 95.3, and 92.237%. From the analysis, it seems that the translator’s identity and the translation era were not a basis for classifying the semantic similarity of the translations. The author’s aim in producing a translation, as well as their intended audience, are important factors in determining translation characteristics. However, based on the analysis of the results of this study, we can see that the different translation purposes do not significantly affect the semantics of the translations. For example, Pound’s main purpose in translating *The Analects* is to interpret the “meaning” of the original work. James Legge translated *The Analects* mainly because he realized in the course of his missionary work that he could only be competent in his duties if he fully mastered the scriptures of the Chinese and personally examined the entire field of thought in which the Chinese sages dabbled in order to explore the foundations of Chinese moral, social, and political life. Although the purposes of the two translators are very different, their semantic similarity is still relatively high.

Through this research, the semantic differences among the classical translations of *The Analects* are found to be mainly due to the use of different Chinese annotations. Some progress is made in the study of the translation of *The Analects* concerning the selection of Chinese annotations. For example, James Legge’s translation in 1861 was based solely on Zhu Xi’s Chinese annotation in the Southern Song Dynasty, wherea D.C. Lau’s translation in 1979 collected the essence of Zhu Xi, He Yan, and Xing Bing’s Chinese annotations and combined them with the translator’s understanding. In 2017, Peimin Ni ‘s translation, in addition to referring to the Chinese annotations of Zhu Xi, He Yan, and Xing Bing and the translator’s understanding, also refers to the research results of Japanese and Korean scholars on the interpretation of the statements of *The Analects*. In China, with the further excavation of archaeological sites, the Chinese annotations on *The Analects* are constantly being optimized, which explains why the above three translators used different Chinese annotations or a collection of several annotations for their translation in different periods. As a result, the author believes that if one wishes to translate The Analects more accurately and rigorously in the future, the process of choosing Chinese annotations must be more rigorous and diversified.

## Conclusion

This study introduces NLP algorithms to translation research and demonstrates how it can be used for the semantic analysis of translated texts, specifically different translations of *The Analects*. For text selection, Python crawler technology is used to obtain a large amount of data, and the research samples are scientifically screened based on data analysis. Through program computation, a quantitative and visual approach is provided. The results show that translations with a better acceptance have a higher semantic similarity, but some differences still remain. As for the analysis of textual differences, through different examples, the authors believe that the translator’s identity, the translation era, and the purpose and characteristics of the translation have no apparent influence on the semantics of the translated text. At the same time, the choice of Chinese annotation is the critical factor affecting the semantics of the translated texts. Furthermore, visually displaying the data of the differences among the translations can trigger scholars to conduct a more in-depth investigation into the various factors that lead to such differences and improve the translated texts.

In the future, translators may study and draw on previous translations of *The Analects*, and this should not be considered an act of plagiarism. Based on previous studies, considering present-day readers’ reading needs, optimizing previous versions by referring to the latest interpretations of the whole or some chapters of *The Analects* will undoubtedly help improve the quality of translations. Meanwhile, when writing translation drafts, translators can use the research method of this study to compare their works with previous translations through semantic similarity detection. They can compare the entire or part of the translated text, focusing on the text with significant semantic differences or parts with semantic anomalies in the draft. In addition, they can determine if translation improvement is needed, thus helping to adjust the translated text and improve the quality of the translation achieve their desired purpose.

In future research, the semantic similarity between the different English translations and the source texts can be further explored, namely, through cross-linguistic comparative evaluation. This function is already possible with the machine translation model using the algorithm in this study. However, the accuracy of machine translation still needs to be improved, and this functional goal cannot be precisely achieved yet. However, the author is sure that, with the continuous improvement of machine translation models, the semantic differences between a translation and the source text can be demonstrated visually by using the NLP algorithm demonstrated in this study, and the semantics of the translation can be evaluated scientifically.

There are also some limitations in this study. For example, this study only obtains data based on five typical representative websites, which can reflect the reader’s acceptance to a certain extent but cannot reflect that of the English translation of *The Analects* comprehensively and accurately. In addition, the author believes that the level of research still needs to be refined, as this study only compares semantics at the macro level and has not yet been able to refine the comparison of semantic differences to the sentence segment level. At the same time, the relevant elements affecting the semantic degree of the translation, such as the identity of the translator, the era of the translation and the degree of influence of the translation purpose on the semantics of the text, also need to be studied at the micro level. Further studies should conduct semantic analyses and comparisons from more English translations of *The Analects* from more microscopic perspectives to explore any semantic differences.

## Data availability statement

The original contributions presented in the study are included in the article/supplementary material, further inquiries can be directed to the corresponding author. Additional data is available via Figshare (https://doi.org/10.6084/m9.figshare.21152092).

## Author contributions

LY and GZ: contributed to conception and design of the study. LY: organized the database, performed the statistical analysis, and wrote the first draft of the manuscript. GZ: gave the paper guidance and some research ideas. All authors contributed to manuscript revision, read, and approved the submitted version.

## Conflict of interest

The authors declare that the research was conducted in the absence of any commercial or financial relationships that could be construed as a potential conflict of interest.

## Publisher’s note

All claims expressed in this article are solely those of the authors and do not necessarily represent those of their affiliated organizations, or those of the publisher, the editors and the reviewers. Any product that may be evaluated in this article, or claim that may be made by its manufacturer, is not guaranteed or endorsed by the publisher.
